# Internal validation study to assess the SeqStudio™ for human identification’s performance

**DOI:** 10.1007/s00414-023-03016-y

**Published:** 2023-05-17

**Authors:** Giulia Soldati, Stefania Turrina, Chiara Saccardo, Francesco Ausania, Domenico De Leo

**Affiliations:** Department of Diagnostics and Public Health, Section of Forensic Medicine, Forensic Genetics Lab, University of Verona, Verona, Italy

**Keywords:** SeqStudio™ Genetic Analyzer for HID, GlobalFiler™ IQC Amplification Kit, DNA typing, Short tandem repeats (STRs), Forensic validation studies

## Abstract

The SeqStudio™ for human identification (HID) is a new benchtop capillary electrophoresis (CE) platform recently developed by Applied Biosystems for genotyping and sequencing short tandem repeat (STR) fragments. Compared to the previous series of CE systems developed by this maker, it is more compact and easier to use. Moreover, by allowing the detection of 4 to 8 fluorescent dyes, it seems to be fully compatible with the different kits of autosomal and gonosomal STR markers usually used in forensic genetics, which are available in trade and supplied by various manufacturers. However, being a new CE model, before its routine use in forensic genetics applications, it should undergo appropriate analytical validation studies in its own laboratories to understand its potential and limitations. A series of experiments on DNA samples coming from cell line controls, using the GlobalFiler™ IQC Amplification Kit, were carried out to meet this purpose. The SeqStudio™ Genetic Analyzer for HID’s findings on genotyping reproducibility (precision and accuracy of sizing), sensitivity, signal variability between dyes (intra- and inter-color channel balance), and stutter ratios are reported. These findings confirm the validity of this new CE system and its capability to generate reliable results.

**Supplementary Information:**

The online version contains supplementary material available at 10.1007/s00414-023-03016-y.

## Introduction

The need to analyze a large number of STR markers (both autosomal and gonosomal) in particular forensic circumstances, to guarantee the accuracy of the acquired genetic profile, as suggested by scientific societies and international government bodies (the European Network of Forensic Science Institutes (ENFSI), the European DNA Profiling Group (EDNAP), and the Federal Bureau of Investigation (FBI)) [1, 2], has prompted forensic kit manufacturers to develop new panels of STR loci by implementing the already existing ones. That has necessitated an inevitable revision of the capillary electrophoresis systems routinely used in forensic genetics laboratories by upgrading the number of fluorophores detectable by the detector and, at the same time, employing particular technical devices, making the instrumentation friendlier for the operator.

An easy-to-use CE benchtop system recently developed by Applied Biosystems is the SeqStudio™ Genetic Analyzer for Human Identification (HID) [3], utilizable for both STR fragments analysis and Sanger sequencing.

Undoubtedly, the presence of a replaceable all-in-one cartridge that incorporates a four-capillary array, a universal POP-1 polymer and its delivery mechanism, and an anode buffer, distinguishes this new version of benchtop CE from the previous series made by Applied Biosystems.

The instrument is set up at the time of use quickly, and its maintenance is easy. Each time the cartridge is installed into the appliance, this automatically performs an optical alignment of each capillary and adjusts the spectral calibration for each sample to correct for dye spectral overlap.

Additionally, thanks to the presence of a detector able to distinguish up to 8 dyes and the data analysis capabilities of GeneMapper ID-X software v1.6, the SeqStudio™ Genetic Analyzer for HID turns out to be compatible with the data interpretation obtained from a variety of STR kits, also provided by different suppliers.

Validation studies have been done by the manufacturer on the SeqStudio™ Genetic Analyzer for HID according to the Scientific Working Group for DNA Analysis Methods (SWGDAM) using the following kits: GlobalFiler, GlobalFiler Express, VeriFiler Plus, VeriFiler Express, Huaxia Platinum, Identifiler Plus, MiniFiler, NGMSelect, NGM Detect, YFiler, and YFiler Plus PCR amplification kits [4].

However, it is a good laboratory practice, before introducing new instrumentation for casework activities, that a laboratory should do internal validation studies to verify if the analytical parameters provided by the appliance meet the ability to ensure proper data interpretation.

This study aims to evaluate the performance of the SeqStudio™ Genetic Analyzer for HID by validating analytical parameters, such as precision, accuracy, resolution, sensitivity, reproducibility, signal variability between dyes (intra- and inter-colour channel balance), and stutter ratios, using the GlobalFiler™ IQC Amplification Kit (Applied Biosystems) on known control DNA samples, enclosed in two STR Kits, at different concentrations.

To reference ease, the abbreviations adopted in the text to define the SeqStudio™ Genetic Analyzer for HID, the GlobalFiler™ IQC Amplification Kit, and Yfiler™ Plus PCR Amplification Kit will be SeqStudio™ for HID, GlobalFiler™ IQC, and Yfiler™ Plus, respectively.

## Materials and methods

### DNA samples

Two DNA control 007 at concentrations of 0.1 ng/μl and 2 ng/μl included as a positive control in the GlobalFiler™ IQC and in the Yfiler™ Plus (Applied Biosystems), and hereafter referred to as DNA control 007-A and 007-B, respectively, were used throughout at a variable range of dilutions to perform the testing in this validation study.

### Precision, accuracy, and resolution

The SeqStudio™ for HID’s run-to-run precision in fragment size calling was assessed using the GlobalFiler™ IQC allelic ladder. Based on the size values observed for each allele in eight runs of the ladder obtained with two successive injections, the mean size (minimum and maximum size) and the standard deviation (SD) for each allele were established.

The accuracy was ascertained by comparing each allele’s dimension in base pair (bp) of 20 single-source profile runs in 5 repeated injections with the corresponding alleles in the allelic ladder. The 20 profiles were obtained by amplifying all serial dilutions of the two DNA control 007-A/B with the GlobalFiler™ IQC at 29 cycles, and for dilutions equal to or below 125 pg/μl, a further amplification was performed at 30 cycles. As passing accuracy, a range of ± 0.5 bp was used.

The SeqStudio™ for HID’s single-base resolution ability was tested on the four GlobalFiler™ IQC loci which exhibit 1-bp-different size alleles (TH01, D2S441, D1S1656, and D12S391). The examination was performed on the eight replicated runs of the GlobalFiler™ IQC allelic ladder, assessing the number of bp that fall into the distance between two size adjacent peaks divided by the peak widths measured at half peak height. The equation proposed by Luckey et al. was applied to calculate the resolution (R) [5].

### Sensitivity

The DNA control 007-A was serially diluted, starting from the initial concentration of 100 pg/μl and producing concentrations of 50, 25, and 12.5 pg/μl; for the DNA control 007-B, dilution series were generated starting at an initial concentration of 2 ng/μl and producing concentrations of 1 ng/μl, 500, 250, 125, 62.5, 32.2, and 15.6 pg/μl. The concentrations up to 62.5 pg/μl were verified with the Qubit Fluorimeter using the Qubit dsDNA HS Assay Kit.

Then, each dilution and non-template control was amplified in triplicate using the GlobalFiler™ IQC kit. The PCR program adopted was the one recommended by the kit’s manufacturer at 29 cycles [6]. Exclusively for the dilutions equal to or below 125 pg/μl, the number of amplification cycles was raised to 30.

Subsequently, each replicate PCR product was typed using the SeqStudio™ for HID, and data analysis was carried out using the GeneMapper ID-X Software v1.6 [7].

### Reproducibility

The reproducibility was tested by comparing the known STR profile of the DNA control 007 with each of the 60 profiles obtained from the serial dilution of the two DNA control 007-A/B, amplified in triplicate with the GlobalFiler™ IQC kit and used to evaluate the sensitivity.

### Intra-locus peak balance, stutter ratios, and signal variability between dyes (intra- and inter-color channel balance)

On STR profiles generated by amplifying the DNA control 007-B at concentrations of 2, 1, and 0.5 ng/μl with the GlobalFiler™ IQC kit, intra-locus peak balance, stutter ratios, and signal variability between dyes were evaluated.

Intra-locus peak balance is the average peak height ratio at a heterozygous locus and was calculated as the percentage ratio of the lower to the upper peak in relative fluorescence unit (RFU) values. Except for the IQCS, IQCL, Yindel, and DYS391 markers, for each locus were determined this parameter, and the peak heights were normalized as follows: homozygous peak heights were divided by two, whereas for heterozygous, the average peak heights were considered.

Minus and plus stutters, as a percentage ratio of the stutter peak height to the main peak height, were calculated, and also the standard deviation was determined.

The intra-color balance was calculated as the percentage ratio between the two loci of the same dye lane having the average lowest and highest peak heights.

Inter-color balance was calculated by the percentage ratio between the total normalized average signal in each color channel and the total normalized signal from all color channels.

### DNA amplification and CE conditions

Each DNA control 007-A/B diluted series was amplified using the GlobalFiler™ IQC following the manufacturer’s instructions [6]. GlobalFiler™ IQC is a kit designed by Applied Biosystems to provide high performance on DNA samples in minimal quantities or a degraded state, having six STR-dyes, five of which are used to label the amplicons of the 24 markers and the two internal quality controls (IQCs), one low molecular weight and the other high molecular weight (IQC-Small and IQC-Large).

The amplified samples were then set up for CE analysis. In each well of a 96-well plate was added 1 μl of each PCR product or allelic ladder, 9.6 μl of deionized Hi-Di Formamide, and 0.4 μl of GeneScan™ 600 LIZ Size Standard v2.0 (Thermo Fisher Scientific), according to the manufacturer recommendations of the STR-kit used [6].

After having made a denaturation at 95 °C for 3 min followed by cooling to 4 °C, the plate was loaded into the autosampler of the SeqStudio™ for HID.

The amplicon separation was performed by SeqStudio™ for HID equipped with a universal all-in-one cartridge consisting of a 4-capillary array 28 cm in length, a POP-1 polymer and its delivery system, and an anode buffer. Every time the cartridge is installed, the SeqStudio™ for HID automatically performs an optical alignment and adjustment of the spectral calibration. In this study, spectral calibration with the DS-36 Matrix Standard was performed. It was to allow the evaluation of the noise levels of six different dyes (Dye Set J6) detectable in every single capillary and to establish a threshold in RFU sufficient to capture the noise generated by the instrument deconvoluting dyes overlap [3].

Since a peak amplitude threshold of 175 RFUs, already defaulted by the manufacturer of SeqStudio™ for HID, has been applied, the presence of pull-up phenomena was visually evaluated in 60 profiles obtained by amplifying all serial dilutions of the two DNA control 007-A/B amplification with GlobalFiler™ IQC in triplicate.

The voltage and time injection (1.2 kV for 15 s), run conditions (13 kV for 1550 s), and run module (HID) were by default performed [3].

### Data analysis

Data collection and fragment analysis were carried out with GeneMapper ID-X v1.6 software (Applied Biosystems) using manufacturer-supplied Panel, Bin, Stutter, and Size Standard files.

## Results

**Fig. 1:**
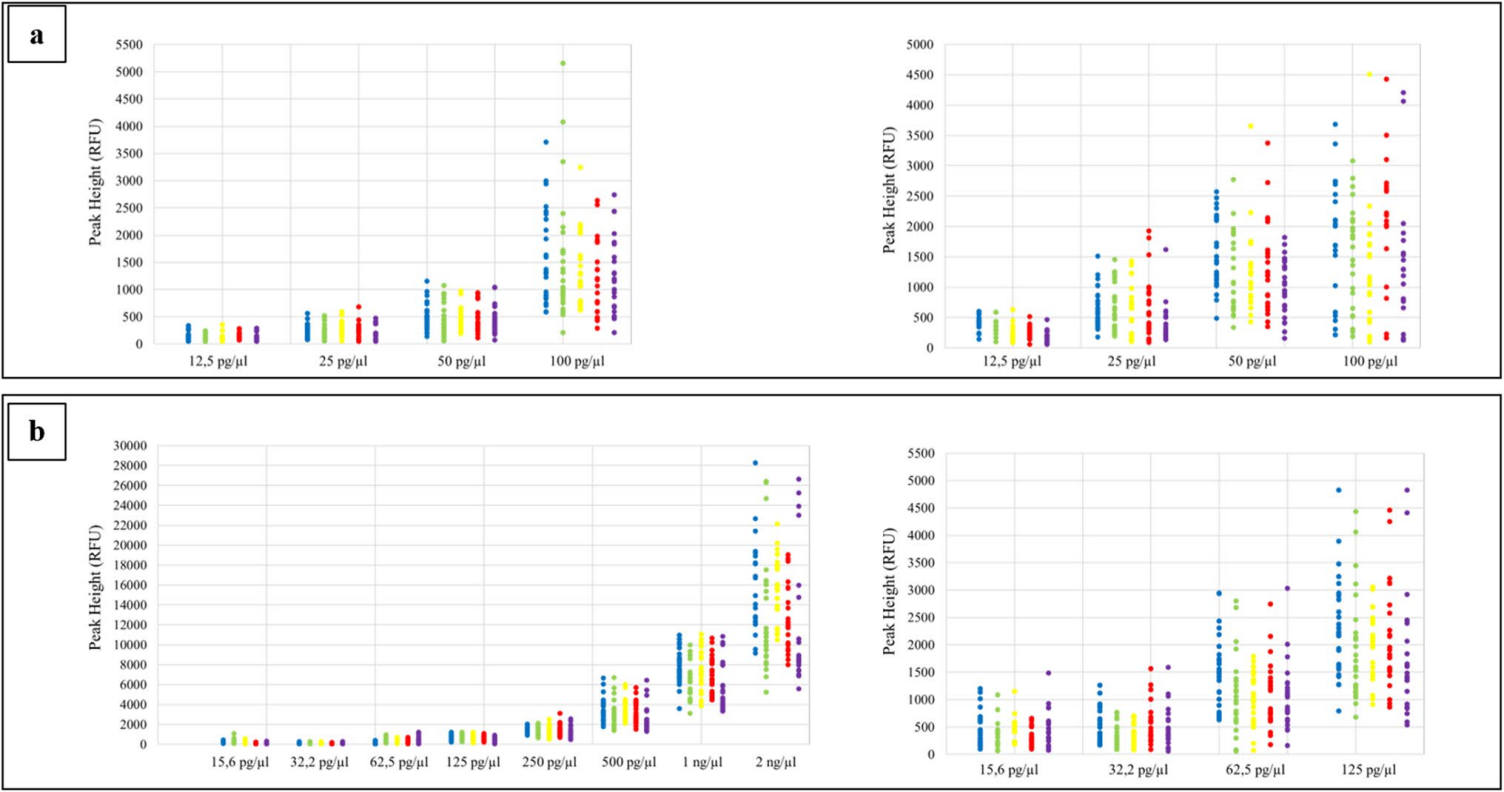
Peak height variability at different concentrations of DNA control 007-A/B (**a**, **b**) at 29 (on the left side) and 30 (on the right side) cycles

The variability of the signal fluorescence intensity generated by each amplicon, obtained with the DNA control 007-A/B serial dilutions at both PCR cycles (29 and 30 cycles), was assessed, finding that there was a direct correlation between the amount of the DNA template used for the PCR reaction and the increase in fluorescence intensity, evaluated as peak height in RFU (Fig. 1).

**Fig. 2:**
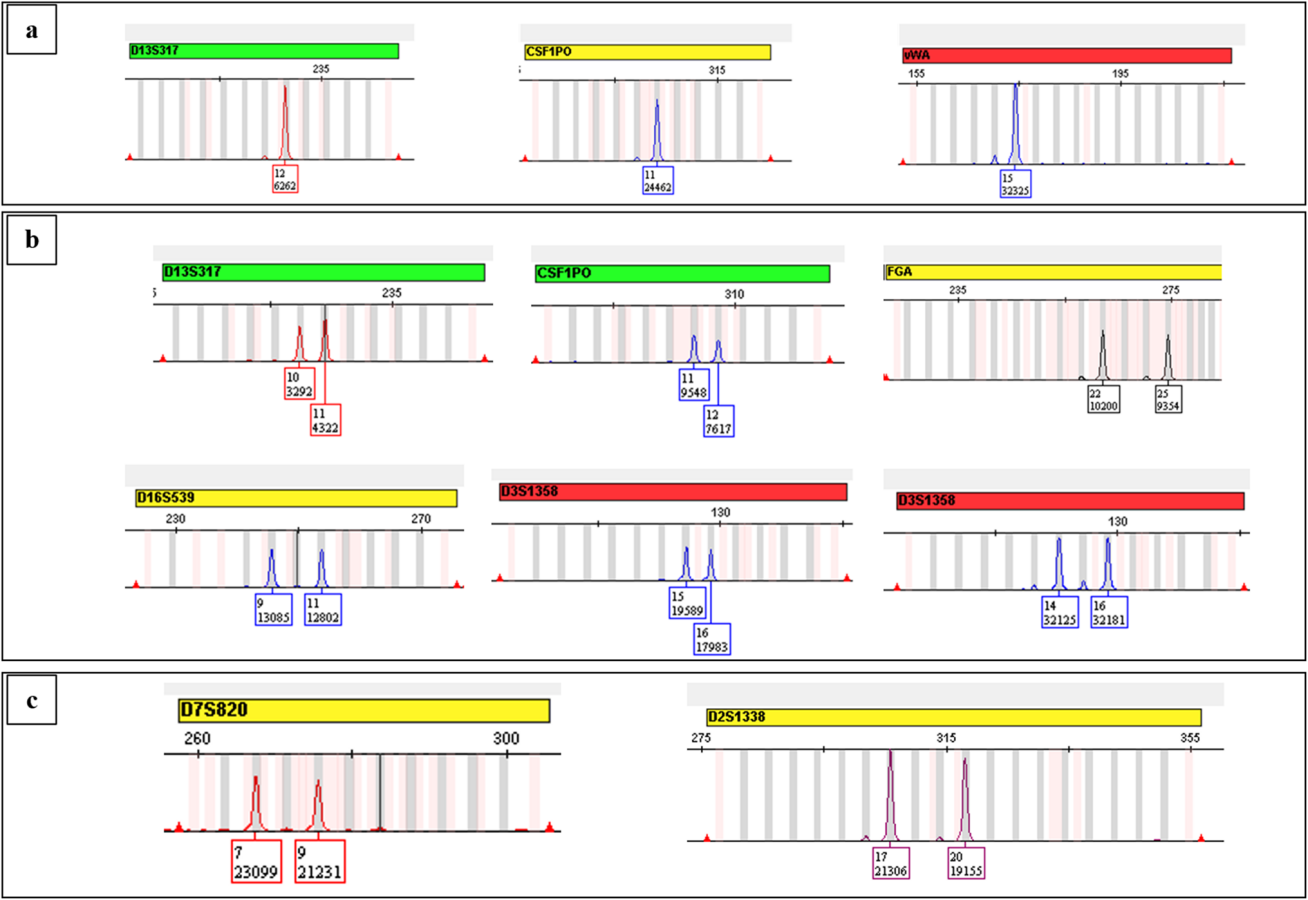
Marker header flagged by GeneMapper ID-X v1.6 in homozygous (**a**) and heterozygous (**b**) genotypes. Unexpected marker header flags were also observed (**c**)

It was noticed that the GeneMapper ID-X v1.6 highlighted the marker header with green, yellow, or red color, mainly depending on the RFU value at the *Y*-axis detected for each peak. The homozygous genotype to a locus is highlighted in green if the single peak height is equal to or less than 10,000 RFU, yellow if it is between 10,000 and 30,000 RFU, and red if the single peak height exceeds 30,000 RFU (Fig. 2a).

The heterozygous balanced genotype to a marker is flagged in green if both the peaks have heights equal to or less than 10,000 RFU, regardless of whether their sum exceeds 10,000 RFU; yellow if one or both peaks have heights > 10,000 RFU, but their sum is less than 30,000 RFU; red if both peak heights are > 10,000 RFU and their sum is more than 30,000; or if one or both peaks have heights > 30,000 RFU (Fig. 2b).

However, it has been observed that sometimes, the software assigns an unexpected color to the marker header, thus disregarding the above. In two distinct profiles, the D7S820 and D2S1338 loci, with heterozygous genotypes, had the heights of both peaks higher than 10,000 RFU with a sum exceeding 30,000 RFU, and therefore, the locus header was expected to be highlighted in red by the software, instead of yellow (Fig. 2c). Given that these genotypes are heterozygous, it is possible to assume that the yellow header of the marker is due to an intra-locus imbalance (< 65%) event; however, even this eventuality should be discarded since the peaks in both loci were properly balanced (> 90%).

### Precision, accuracy, and resolution

Run-to-run precision obtained on data collected from eight runs performed in two successively repeated injections of the GlobalFiler™ IQC allelic ladder provided a maximum SD value of 0.083 bp and a maximum difference range in size of 0.26 bp (Supplementary Table S1).

**Fig. 3:**
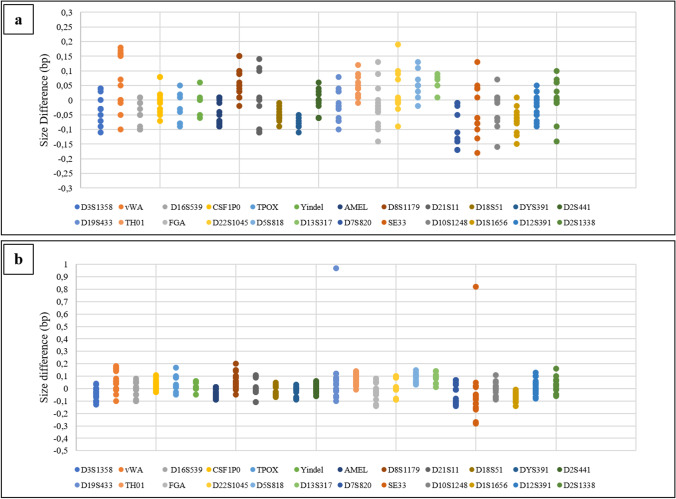
Allele accuracy results for DNA control 007-A/B (**a**, **b**) detected on the SeqStudio™ for HID. The size difference (bp) is in the range of ± 0.2 bp for each locus, with the exception of SE33 and D19S433 markers in the DNA control 007-B (**b**)

For twenty DNA control 007-A/B profiles, comparing each allele with the corresponding one in the allelic ladder, the accuracy of alleles resulted to be in the range of ± 0.2 bp (Fig. 3).

The only exception was observed in two dilutions of DNA control 007-B at 15.6 pg/μl and 0.5 ng/μl amplified at 29 cycles, in which the size comparison between the sample’s allele and the allelic ladder for the loci SE33 and D19S433 resulted in 0.82 bp and 0.97 bp of difference, respectively (Fig. 3b). When these two dilutions were amplified at 30 cycles, the accuracy was again in the range of ± 0.2 bp.

**Fig. 4:**
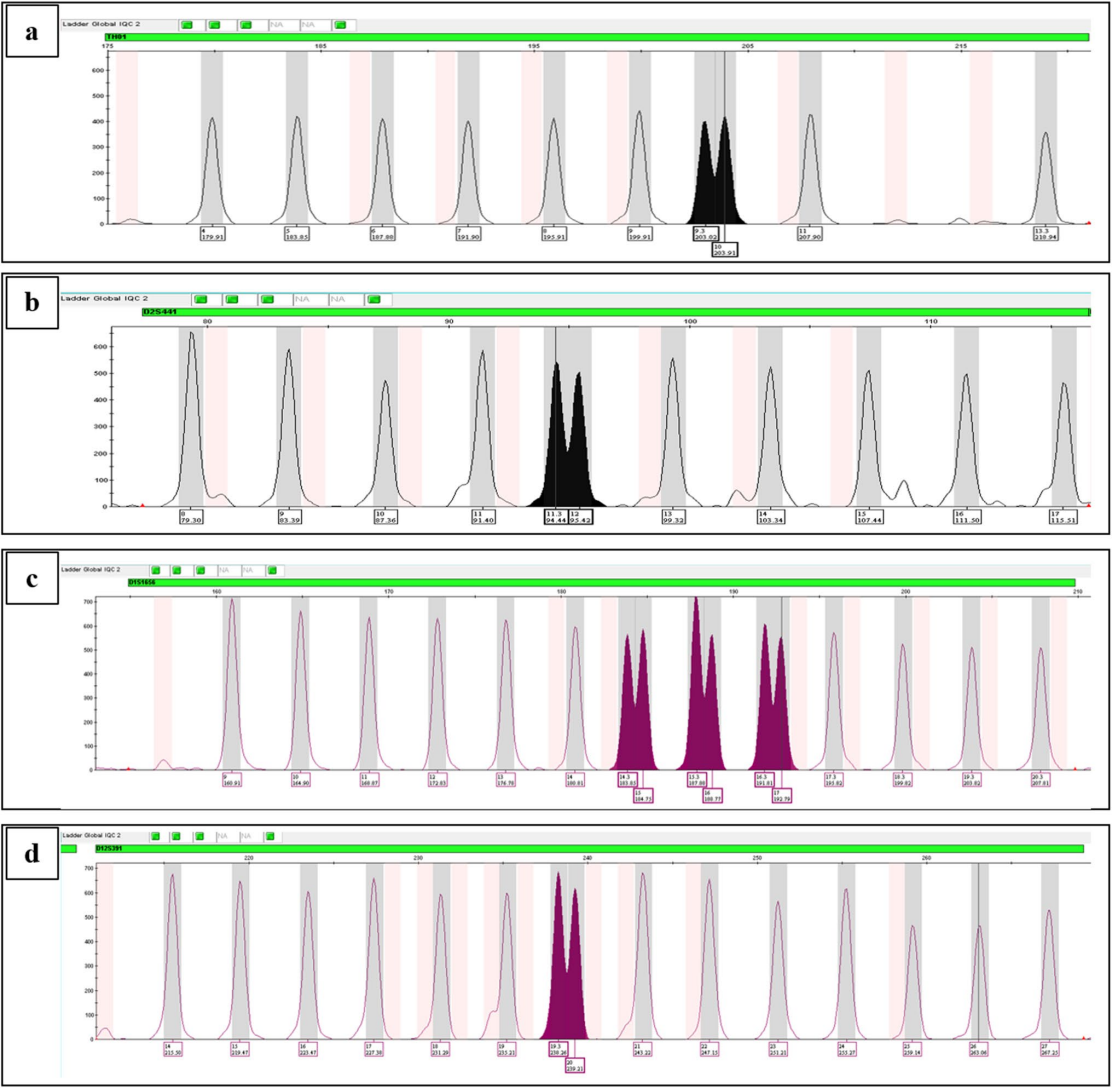
GlobalFiler™ IQC allelic ladder resolution evaluated on the SeqStudio™ for HID at TH01 (**a**), D2S441 (**b**), D1S1656 (**c**), and D12S391 (**d**) loci

In the eight replicate runs of the GlobalFiler™ IQC allelic ladder, the resolution was assessed for alleles separated by 1 bp at the TH01, D2S441, D1S1656, and D12S391 loci (Fig. 4). *R*-values were between 1.01 and 1.32, with mean values ranging from 1.15 to 1.49 (Supplementary Table S2).

### Sensitivity

The sensitivity of the SeqStudio™ for HID was tested on a range of serial dilutions of the two DNA control 007-A/B using a 175 RFU peak detection threshold.

Full profiles and precision in allele calling were observed in all replicates for the DNA dilutions ranging from 2 ng/μl to 125 pg/μl amplified at 29 and 30 cycles.

At the input DNA concentration of 2 ng/μl, in the three replicates, off-scale peaks were observed at all loci, regardless of homozygous or heterozygous genotype, with the only exception at the heterozygous markers D7S820, D10S1248, and D1S1656, in the red and purple channels, respectively. Although in the heterozygous genotypes, the off-scale peaks were expected, as the DNA concentration was higher than recommended for amplification, the peaks still remained quite well balanced.

At 1 ng/μl DNA control 007-B, in the three replicates, on-scale peaks in the majority of the loci, whether the homozygous or heterozygous genotype, were detected. Exceptions were revealed in the blue channel at the heterozygous D3S1358 in one replicate and the homozygous TPOX in two replicates; in the yellow channel at the heterozygous D2S441 in two replicates; in the red channel at D5S818 and D13S317 loci, both in homozygosis, in a single replicate; in the purple channel at the heterozygous D2S1338 in two replicates, where the software highlighted in yellow the header of these loci because the peak heights exceeded 10,000 RFU.

At concentrations of 500, 250, and 125 pg/μl, in all replicates, on-scale peaks at all loci occurred, whether the homozygous or heterozygous genotype. However, the number of loci detected by the software showing heterozygous imbalance (< 65%) [6] increased as progressive dilution of the DNA control 007-B.

At concentrations below 125 pg/μl, although in all replicates on-scale peaks at all loci were revealed, genetic profiles characterized by false homozygous conditions due to allelic drop-outs and partial profiles due to the lack of genotyping of some loci were observed. Specifically, at the concentration of 100 pg/μl in two separate replicates at 30 cycles, a state of false homozygosity for D12S391 and D2S1338 markers was found. At DNA concentrations of 62.5 and 50 pg/μl (29–30 cycles), false homozygosity was also detected at other loci.

At concentrations equal to or less than 32.25 pg/μl, false homozygosity in half of the loci and partial profiles due to full drop-out was found (Supplemental Fig. S1).

### Reproducibility

Reproducibility was assessed by comparison of the known STRs profile of the DNA control 007 with the genetic profiles obtained in triplicates with both DNA control 007-A/B serial dilutions. While at DNA concentrations equal to or greater than 125 pg/μl reproducibility of the STR profiles was 100%, it decreased with decreasing DNA concentration used in PCR amplification, regardless of the number of PCR cycles employed. At DNA concentrations equal to or below 62.5 pg/μl, the STR profiles were characterized by partial and full allele drop-out events.

### Intra-locus, intra-color, and inter-color balance

The intra-locus balance and intra- and inter-color balance percentages were calculated on three triplicates of the DNA control 007-B at 2, 1, and 0.5 ng/μl concentrations.

**Fig. 5:**
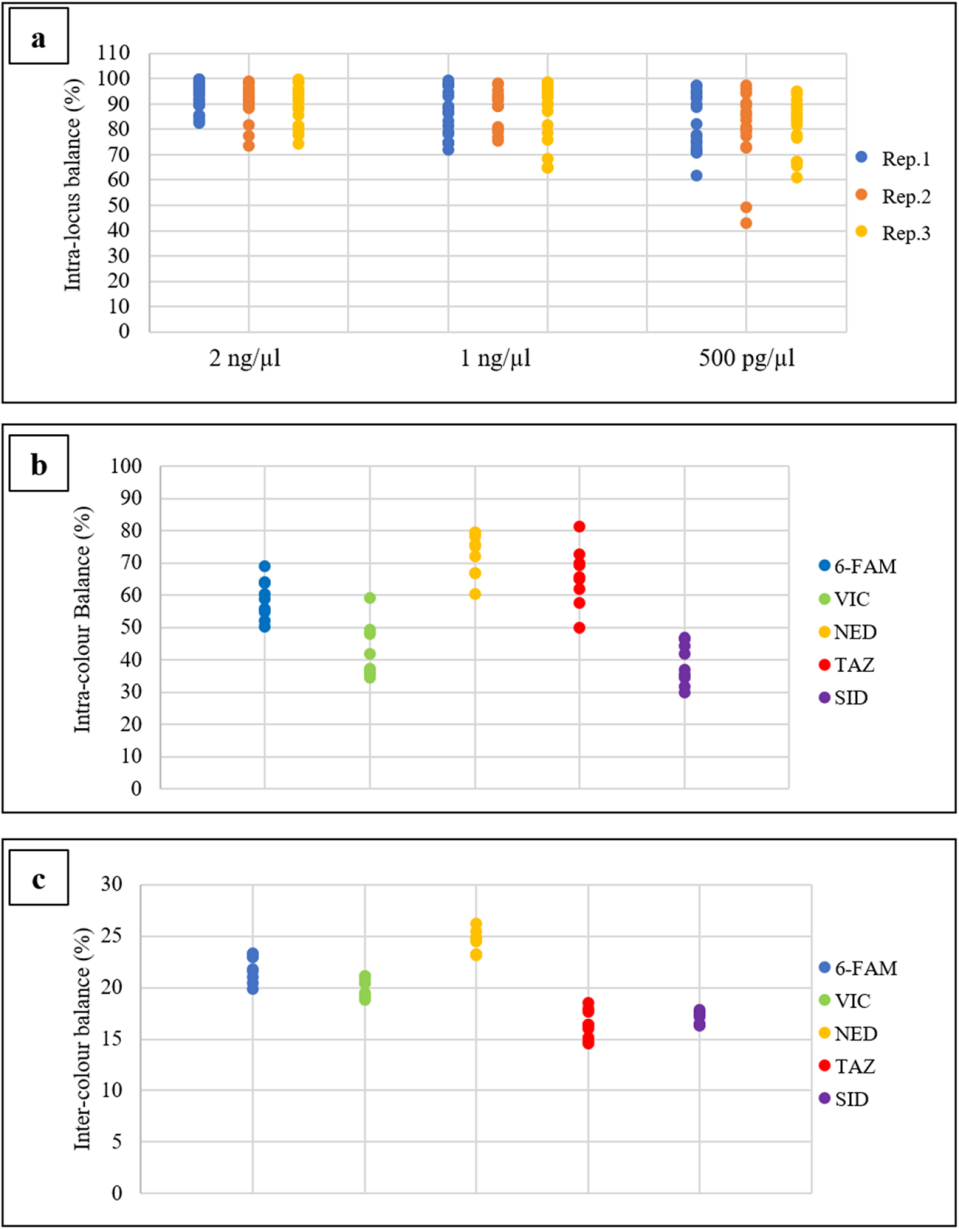
Intra-locus balance (%) (**a**), intra-color balance (%) (**b**), and inter-color balance (%) (**c**) for DNA control 007-B at concentrations of 2, 1, and 0.5 ng/μl

For heterozygous loci, intra-locus balance values > 65% [6] were obtained in all replicates. Some exceptions were detected at DNA concentrations of 1 and 0.5 ng/μl, where some loci had intra-locus balance in the range of 40–65% (Fig. 5a).

In all replicates made at the three different concentrations of the DNA control 007-B, the loci had intra-color balance values equal to or greater than 40% [6] in the blue, yellow, and red channels. For the green and purple channels, at 2 ng/μl, all replicates were unbalanced (< 37%); at 1 and 0.5 ng/μl, in a single replicate, intra-color imbalances were below 36% (Fig. 5b).

A profile is considered completely balanced when the inter-color balance percentage ratio is equal to or higher than 20% of the total signal [6]. The inter-color balance of the blue and yellow dyes was consistently greater than 20%; the green dye was imbalanced in one replicate at DNA concentrations of 2 and 0.5 ng/μl and in two repetitions at 1 ng/μl; the red and purple dyes constantly generated unbalanced values in the range of 15–18% (Fig. 5c).

### Stutter effect and cross-channel pull-up

To identify the minus and plus stutters from true alleles, the mean percentage value of each stutter peak was calculated. At loci D22S1045 and SE33 (trimeric and tetrameric markers, respectively), non-standard stutter peaks in the form of n-2 and n ± 3 nucleotides from the target allele, respectively, were observed.

The mean stutters for each locus plus three standard deviations, i.e., stutter percentage filter [8–11], obtained in this study, agree with those reported by the GlobalFiler™ IQC manufacturer as determined using a different CE instrument, with the only exception of TPOX and TH01 loci (Supplementary Table S3).

Cross-channel pull-ups were never detected, not even when the input DNA concentration was higher than recommended for performing PCR (2 ng/μl).

## Discussion

A validation study within a laboratory for a modified technology usable in forensic casework aims to ascertain that the analytical parameters that ensure the reproducibility and reliability of the technology results adhere to pre-established guidelines (SWGDAM) and declared by the manufacturer [3, 4].

Recently, Applied Biosystems has developed a new benchtop CE platform — SeqStudio™ Genetic Analyzer for Human Identification (HID) — that is more compact in dimensions and easier to use than previous CE versions. The ability of the SeqStudio™ for HID to analyze up to 8 dyes makes it compatible with all commercially available autosomal and gonosomal STR kits, but above all, it features a replaceable cartridge, storable at 2–8 °C when not in use, containing a capillary array, a polymer, and its delivery system, and the anode buffer. The presence of an all-in-one cartridge simplifies the instrumentation’s assembly, making the operations extremely quick. Additionally, owing to an intuitive interface for the user, from the instrument’s touch screen, it may be picked to run setup, re-runs, and strip setup changes, as well as view all consumables and expiration dates.

Certainly, the innovative aspects of the SeqStudio™ for HID are the new universal polymer, POP-1, which allows simultaneous DNA fragment analysis and Sanger sequencing on the same injection without the need to use other polymer types and the reduction in capillary length from 36 to 28 cm. However, these changes may affect the performance of the new CE system, particularly data reproducibility. Therefore, this internal validation study on SeqStudio™ for HID aimed to evaluate parameters such as precision, accuracy, resolution, sensitivity, reproducibility, intra-locus balance, stutter effects, and signal variability between dyes, using data obtained from DNA amplification with GlobalFiler™ IQC and analysis with GeneMapper ID-X v1.6 software.

Microvariant alleles that differ in size by a single nucleotide can be effectively distinguished when the DNA sizing precision from run-to-run in several injections falls below a standard deviation of 0.15 bp [8]. Based on the SD obtained in this internal validation study (SD < 0.083 bp), it is possible to state that SeqStudio™ for HID has a resolution capability lower than 1 bp, thereby meeting the performance criteria.

The allele size range of ± 0.5 bp per injection was not exceeded, as the obtained accuracy findings were all within ± 0.2 bp, ensuring an accurate calling of even microvariant alleles by the SeqStudio™ for HID. However, it was observed that if the amplification of low input DNA concentrations is performed out at 30 cycles instead of 29 cycles, this allows achieving the accuracy range mentioned above, also for those loci (SE33 and D19S433) that had greater accuracy values.

The STR markers used for individual identification are generally characterized by tetranucleotide repeats, which, however, do not exclude the presence of allelic variants that differ from each other by a single base. Having a system capable of resolving alleles that differ by a single base ensures the correct allele calling for each detected variant. The SeqStudio™ for HID, in the eight replicated runs of the GlobalFiler™ allelic ladder, was able to ensure a well 1-bp peak resolution for the four TH01, D2S441, D1S1656, and D12S391 loci exhibiting alleles that differ by a single base, with *R* values in the interval ranging from 1.15 to 1.49.

Full profiles and allele calling accuracy were observed in all replicates for DNA dilutions ranging from 2 ng/μl to 125 pg/μl, amplified at 29 and 30 cycles.

At an input DNA concentration of 2 ng/μl, as expected, since this concentration was higher than that recommended for PCR amplification, off-scale peaks were detected at all loci but without pull-up phenomena in the different dye channels. To the other input DNA concentrations, on-scale peaks without heterozygous imbalance were detected.

At input DNA concentrations below 125 pg/μl, false homozygosity due to allelic drop-out and partial profiling due to lack of genotyping of some loci has been observed. These events became more evident at concentrations between 100 and 50 pg/μl, affecting half of the loci at concentrations equal to or lower than 32.25 pg/μl, confirming that the presence of allelic drop-outs in the profiles is related to the input DNA concentration.

Reproducibility of known genetic profiles occurred in the range of 2 ng/μl–125 pg/μl.

Regarding the intra-locus balance, most of the heterozygous loci had balanced peak heights in all three replicates (> 65%), anyway, highlighting that the decrease of DNA inputs influences this parameter, resulting in less balanced.

Setting a value of 40% as the intra-color balance threshold, it was observed that the concentration of input DNA influences this parameter. In particular, at a concentration of 2 ng/μl, higher than the one recommended by the GlobalFiler™ IQC manufacturer, the imbalance mainly concerned the green and purple channels.

Assuming that a profile is considered fully balanced when the inter-color balance percentage ratio is equal to or greater than 20% of the total signal, inter-color imbalances were detectable in the green dye most frequently at DNA input concentrations of 1 ng/μl and consistently found in the red and purple channels.

With the only exception of the TPOX and TH01 loci, the stutter percentage filter obtained in this study agreed with those reported by the GlobalFiler™ IQC manufacturer as determined using a different CE instrument.

One concern does not relate to the performance of the SeqStudio™ for HID but rather the cost of the consumables. Specifically, the all-in-one cartridge of the SeqStudio™ for HID is programmed to carry out, in a maximum period of 6 months after the first installation in the instrument, up to 250 injections, which, considering the four-capillary array, correspond to 1000 typable samples. In routine laboratory practice, however, it is not always possible to integrate four reactions with a single injection; sometimes, the samples to be typed are fewer than four.

Failure to match 250 injections versus 1000 samples could affect instrument management costs by necessitating more frequent cartridge purchases than planned.

## Conclusions

The analytical parameters obtained and evaluated in this internal validation study conducted using GlobalFiler™ IQC on two serial dilutions of DNA control 007 from two STR kits demonstrate that the SeqStudio™ Genetic Analyzer for HID can provide reliable and reproducible data, meeting the guidelines for the validation of DNA analysis methods, as declared by the manufacturer.

## Supplementary information

ESM 1: **Supplemental figure S1** Sensitivity results for serial dilutions of the two DNA control 007-A/B (**a**, **b**). Three replicates of GlobalFiler™ IQC were run over a range of DNA control 007-A/B input amounts (y-axis). Green= properly alleles called; Yellow= only one of the two expected alleles in heterozygous genotype was called; Red= no alleles called.

ESM 2: Table S1 Precision results of the SeqStudio™ for HID using GlobalFiler™ IQC allelic ladder. For each locus, the allele, the average minimum and maximum size value in bp, the Standard Deviation (SD), and the allele size range in bp are reported. Table S2 Allele that differer in 1-bp resolution at TH01, D2S441, D1S1656, and D12S391 loci. Table S3 Stutter mean percentages, minimum and maximum stutter percentages, standard deviations (SD), and stutter filter for the GlobalFiler™ IQC marker set on the SeqStudio™ for HID. The sample set was DNA control 007-B at concentrations of 2, 1, and 0.5 ng/μl
