# Correction to: Internal validation study to assess the SeqStudio™ for human identification’s performance

**DOI:** 10.1007/s00414-023-03071-5

**Published:** 2023-08-03

**Authors:** Giulia Soldati, Stefania Turrina, Chiara Saccardo, Francesco Ausania, Domenico De Leo

**Affiliations:** Department of Diagnostics and Public Health, Section of Forensic Medicine, Forensic Genetics Lab, University of Verona, Verona, Italy

**Correction to: International Journal of Legal Medicine (2023) 137:971–980**

**https://doi.org/10.1007/s00414-023-03016-y**

Originally, the online published article contains an inverted author names. Family name was captured first instead of the given names. This is now updated here.

The original article has been corrected.

