# Supplementary material for: Internal validation study to assess the SeqStudio™ for human identification’s performance
Source: Int J Legal Med. 2023 May 17;137(4):971–80. doi: 10.1007/s00414-023-03016-y (PMC10247830; doi:10.1007/s00414-023-03016-y)
Supplement: Supplementary file 1 — Supplemental figure S1 Sensitivity results for serial dilutions of the two DNA control 007-A/B (a, b). Three replicates of GlobalFiler™ IQC were run over a range of DNA control 007-A/B input amounts (y-axis). Green= properly alleles called; Yellow= only one of the two expected alleles in heterozygous genotype was called; Red= no alleles called. [file 414_2023_3016_MOESM1_ESM.docx]

**Internal validation study to assess the SeqStudio™ for Human Identification’s performance**

*International Journal of Legal Medicine*

Soldati Giulia^1^, Turrina Stefania^1^, Saccardo Chiara^1^, Ausania Francesco^1^, De Leo Domenico^1^

^1^ Department of Diagnostics and Public Health, Section of Forensic Medicine, Forensic Genetics Lab, University of Verona, Italy

Corresponding author: [giulia.soldati_02@univr.it](mailto:giulia.soldati_02@univr.it), ORCID iD 0000-0001-7442-8139


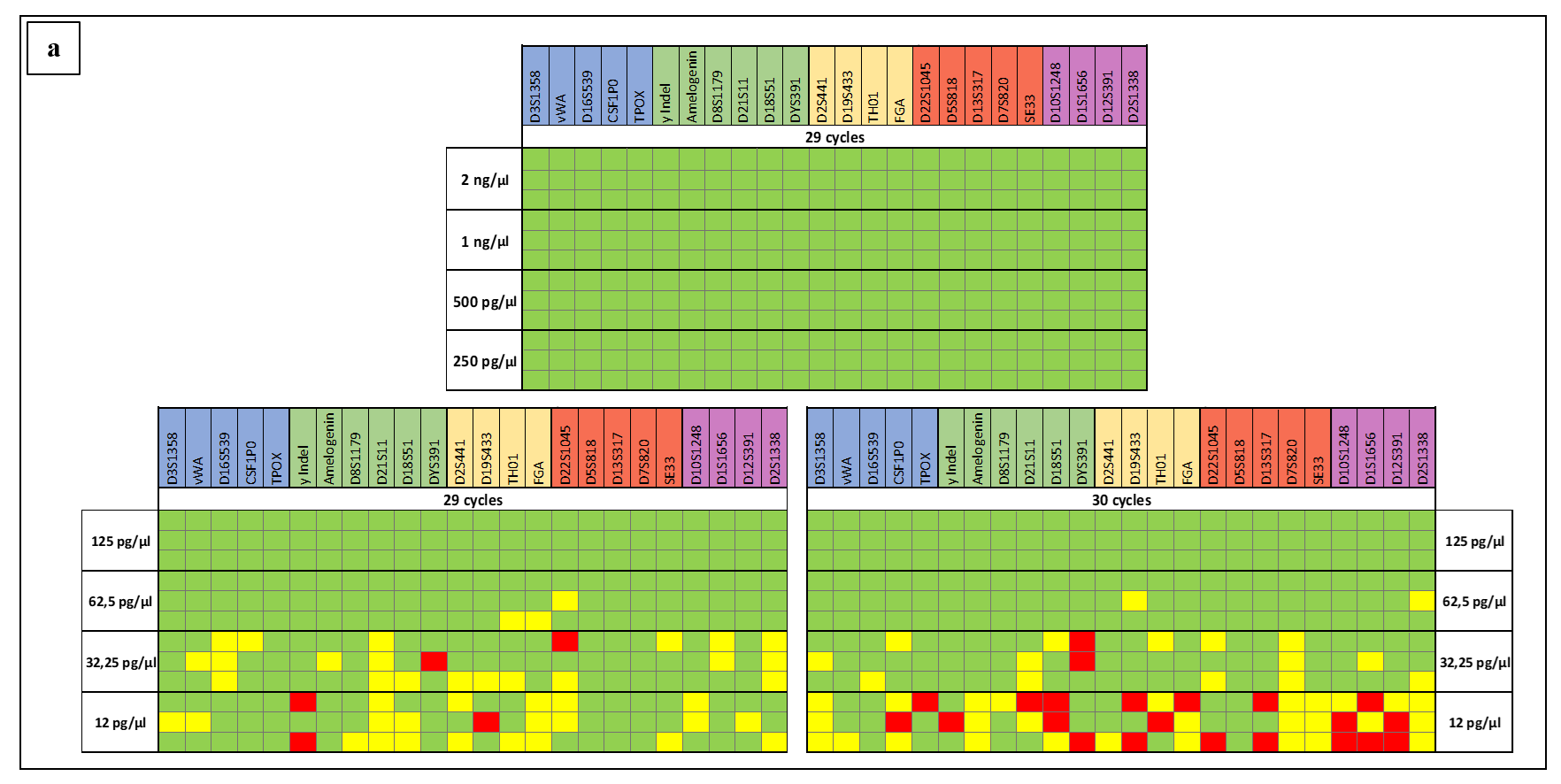

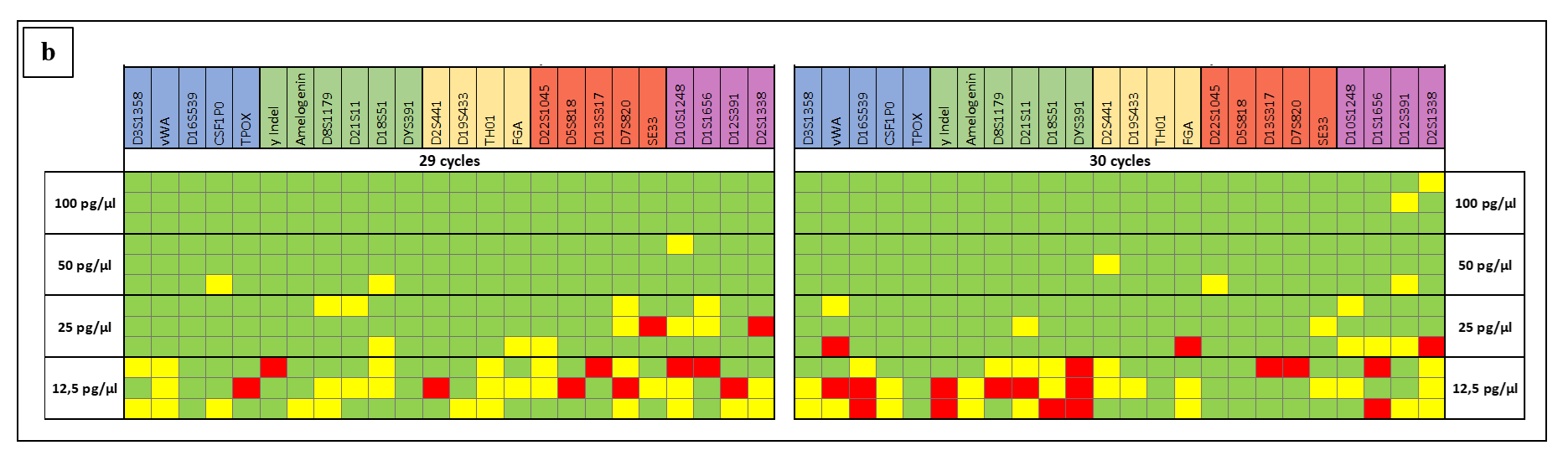


**Supplemental figure S1** Sensitivity results for serial dilutions of the two DNA control 007-A/B (**a**, **b**). Three replicates of GlobalFiler™ IQC were run over a range of DNA control 007-A/B input amounts (y-axis). Green= properly alleles called; Yellow= only one of the two expected alleles in heterozygous genotype was called; Red= no alleles called
